# Epstein–Barr virus microRNAs and lung cancer

**DOI:** 10.1038/bjc.2011.221

**Published:** 2011-06-07

**Authors:** J Koshiol, M L Gulley, Y Zhao, M Rubagotti, F M Marincola, M Rotunno, W Tang, A W Bergen, P A Bertazzi, D Roy, A C Pesatori, I Linnoila, D Dittmer, A M Goldstein, N E Caporaso, L M McShane, E Wang, M T Landi

**Affiliations:** 1 Infections and Immunoepidemiology Branch, Division of Cancer Epidemiology and Genetics, National Cancer Institute, National Institutes of Health, Department of Health and Human Services, Bethesda, MD, USA; 2 Department of Pathology and Laboratory Medicine and the Lineberger Cancer Center, University of North Carolina at Chapel Hill, Chapel Hill, NC, USA; 3 Biometric Research Branch, Division of Cancer Treatment and Diagnosis, National Cancer Institute, National Institutes of Health, Department of Health and Human Services, Bethesda, MD, USA; 4 EPOCA Epidemiology Research Center, Department of Occupational and Environmental Health, Università degli Studi di Milano, Milan, Italy; 5 Epidemiology Unit, Fondazione IRCCS Ospedale Maggiore Policlinico, Mangiagalli e Regina Elena, Milan, Italy; 6 Infectious Disease and Immunogenetics Section (IDIS), Department of Transfusion Medicine, Clinical Center and Center for Human Immunology (CHI), National Institutes of Health, Department of Health and Human Services, Bethesda, MD, USA; 7 Genetic Epidemiology Branch, Division of Cancer Epidemiology and Genetics, National Cancer Institute, National Institutes of Health, Department of Health and Human Services, Bethesda, MD, USA; 8 Center for Health Sciences, SRI International, Menlo Park, CA, USA; 9 Department of Microbiology and Immunology, University of North Carolina at Chapel Hill, Chapel Hill, NC USA; 10 National Institutes of Health Clinical Center, National Institutes of Health, Department of Health and Human Services, Bethesda, MD, USA

**Keywords:** Epstein–Barr virus, lung cancer, microRNA, microarray, qPCR

## Abstract

**Background:**

We conducted the first analysis of viral microRNAs (miRNAs) in lung cancer, with a focus on Epstein–Barr virus (EBV).

**Methods:**

We evaluated viral miRs with a two-channel oligo-array targeting mature, anti-sense miRNAs in 290 cases. In 48 cases, we compared microarray and real-time quantitative PCR (qPCR) expression for three EBV miRNAs. We tested for EBV DNA, RNA, and protein in tumour tissue from six cases with and six cases without strong qPCR-based evidence of EBV miRNAs.

**Results:**

The EBV miRNAs strongly differentiated between adenocarcinoma and squamous cell carcinoma using the microarray (*P*<0.01 for 9 out of 16 EBV miRNAs). However, microarray and qPCR measurements of BART1, BART2, and BHRF1–3 expression were not significantly correlated (*P*=0.53, 0.94, and 0.47, respectively). Although qPCR provided substantial evidence of EBV miRNAs in 7 out of 48 cases, only 1 of these 7 cases had detectable EBV DNA in tumour tissue. None had detectable EBV RNA or protein by histochemical stains.

**Conclusion:**

In a comprehensive evaluation of EBV miRNA, DNA, RNA, and protein in lung cancer, we found little evidence of EBV in lung tumour tissue. Discrepancies between microarray- and qPCR-based strategies highlight the difficulty of validating molecular markers of disease. Our results do not support a role of EBV in lung cancer.

Lung cancer is the most common cause of cancer death in the world (Parkin *et al*, 2005). Although smoking is the primary aetiologic agent, only 10–20% of smokers develop lung cancer (Thun *et al*, 2002), and other cofactors that influence susceptibility are an area of intense investigation. In addition, lung cancer is estimated to be the seventh most common cause of cancer death worldwide in never smokers, a population in which risk factors for lung cancer are not well understood (Sun *et al*, 2007).

Infectious agents are hypothesised to contribute to lung cancer carcinogenesis (Engels, 2008). This hypothesis may be most plausible for lung adenocarcinoma, which occurs more often at younger ages and in never smokers and women than squamous cell carcinoma, and is histologically similar to ovine pulmonary adenocarcinoma, a type of lung cancer in sheep caused by jaagsiekte sheep retrovirus (Sun *et al*, 2007). This example of viral-related lung cancer in sheep suggests that viruses might also contribute to lung carcinogenesis in humans.

Epstein–Barr virus (EBV), a herpesvirus classified as a group 1 carcinogen by the International Agency for Research in Cancer (Bouvard *et al*, 2009), has been proposed as a risk factor for lung cancer. Epstein–Barr virus is clearly associated with lymphoma and nasopharyngeal carcinoma (Kutok and Wang, 2006). It has also been implicated in ∼10% of gastric cancers (Young and Rickinson, 2004). Although lung cancer cases in serological studies had higher EBV seropositivity than healthy controls (Desgranges and de-The, 1979; Roy *et al*, 1994), previous studies of EBV in lung tumour tissue have generally produced negative results, except in rare cases of lymphoepithelioma-like carcinomas (LELCs) (Chu *et al*, 2004; Lim *et al*, 2009). However, previous studies largely tested for expression of EBV-encoded small RNA (*EBER*), which is generally abundantly expressed in cells with latent EBV infection but occasionally may be absent or heterogeneously expressed in EBV DNA-positive tumours (Iwakiri and Takada, 2010).

A novel way to assess the involvement of infections like EBV is to look for viral microRNAs (miRNAs) (Lagana *et al*, 2010). The miRNAs are small, single-stranded RNAs that regulate gene translation and are involved in many biological processes, including immune system development and inflammatory responses (Schetter *et al*, 2010). Viral miRNAs regulate viral gene expression and may also affect the expression of host genes, such as those involved in cellular proliferation and apoptosis, thereby potentially affecting cancer development and progression (Lagana *et al*, 2010). Given the stability of miRNAs even in archival tissue (Koshiol *et al*, 2010), miRNA expression might be a useful way to screen for cancer-related infections.

To our knowledge, no previous study has tested for viral miRNA expression in lung cancer. We evaluated viral miRNA expression in the Environment And Genetics in Lung cancer Etiology (EAGLE) study using two independent viral miRNA expression assays: a miRNA microarray chip and real-time quantitative PCR (qPCR). However, results from different miRNA expression platforms are not always consistent using the same biologic specimens (Koshiol *et al*, 2010). In addition, viruses like EBV may be present in the infiltrating lymphocytes rather than the tumour cells themselves. Thus, we also tested for EBV DNA and looked for histochemical evidence of viral RNA and protein localisation to the tumour cells or infiltrating lymphocytes of lung cancer cases.

## Materials and methods

### Study population

As previously described (Landi *et al*, 2008), EAGLE is a population-based case–control study of 2100 consecutive incident lung cancer cases and 2120 age-, sex-, and residence-frequency-matched population controls, all Caucasians, enroled from 2003 to 2005 in northern Italy. Institutional Review Boards at the National Cancer Institute and all other participating institutes gave approval, and each subject signed an informed consent form. Paraffin-embedded tissue blocks were available for 656 patients. Patients who received chemotherapy or radiation therapy before tissue collection, had tumours with mixed histologies or histologies other than adenocarcinoma or squamous cell carcinoma, or had insufficient tumour tissue were excluded. The current study included all remaining adenocarcinoma (*N*=165) and squamous cell carcinoma cases (*N*=125) who had sufficient tumour tissue available (Landi *et al*, 2010).

### Laboratory methods

#### Microarray (*N*=290)

The miRNA expression in adenocarcinoma and squamous cell carcinoma cases was compared using a custom-made two-channel oligo-array of 713 human, mammalian, and viral mature antisense miRNAs including 2 internal controls with 7 serial dilutions starting at 5 *μ*M and ending at a final dilution of 0.0016 *μ*M (http://madb.nci.nih.gov/gal_fi les/CCDTM-miRNA700-V3px-A.gal). The miRNA chip included miRNAs for EBV (*N*=21), human cytomegalovirus (HCMV; *N*=8), Kaposi's sarcoma-associated herpes virus (KSHV, or human herpesvirus 8; *N*=12), and simian virus 40 (SV40; *N*=1). FlashPage Enriched small RNA, equivalent to 10 *μ*g of the total RNA from formalin-fixed paraffin-embedded tissue blocks, and EBV reference samples spiked in with 2.5 *μ*M antisense control oligos were labelled with Cy5 and Cy3, respectively, using *mir*Vana miRNA Labelling Kit (Ambion, Austin, TX, USA) (Landi *et al*, 2010). The microarray-based expression ratio for each miRNA was normalised by dividing it by the median ratio computed over all miRNAs on the microarray. This method was selected on the basis of a separate study that directly compared the performance of several miRNA microarray normalisation methods (Zhao *et al*, 2010). Additional details and results of the human miRNA analyses are published elsewhere (Landi *et al*, 2010). Missing microarray data can arise when the background intensity is greater than the foreground intensity or when both signals on a dual-channel array are below the specified minimum threshold (set to 100, as per common practice; Simon *et al*, 2004), potentially leading to high levels of missing data. Thus, analyses included only miRNAs with <50% missing data across all 290 samples.

#### qPCR (*N*=48)

Using previously described qPCR assay methods (O’Hara *et al*, 2008; Xia *et al*, 2008), we attempted to confirm selected microarray results. We focussed on EBV miRNAs, which produced many of the greatest differences in expression by histology. We selected the EBV BART1 and BART2 pre-miRNAs because the assays for these two miRNAs were well-validated and because these pre-miRNAs are further processed to make multiple individual EBV miRNAs that had been targeted by the microarray. We also targeted the BHRF1–3 pre-miRNA as it is representative of the miRNAs encoded by a different portion of the EBV genome. We chose 48 RNA samples (25 from adenocarcinoma, 23 from squamous cell carcinoma) with sufficient material for confirmation of viral and human miRNA expression as previously described (Landi *et al*, 2010). Of each sample, 5 *μ*l was diluted into 40 *μ*l with RNase-free water. First-strand cDNA was synthesised from 20 *μ*l RNA using SuperScript First-Strand Synthesis System for qPCR (Invitrogen, Carlsbad, CA, USA). The EBV BART1, BART2, and BHRF1–3 primers targeting EBV pre-miRNAs were applied in replicate with four replicate amplifications per primer pair, for a total of 12 results in each of 48 specimens, or 576 test results. For each specimen, the average number of detectable results was calculated. Specimens with ⩾50% EBV miRNA positivity (at least 6 out of 12 amplifications with detectable EBV miRNA) were considered to have sufficient evidence for the presence of EBV miRNA. Primers targeting miR-16 were chosen as an internal control for miRNA preservation, quality, and amplifiability because it is expressed consistently in human tissues (Liang *et al*, 2007). All qPCR assays were conducted without knowledge of microarray results. Adequate miRNA recovery was confirmed in 46 out of 48 paraffin-embedded tissues.

#### Assessment of EBV localisation (*N*=12)

As the EBV miRNA signal could conceivably come from infiltrating lymphocytes, histochemical analyses were conducted in tumour tissues from all cases with sufficient evidence of EBV miRNAs as measured by qPCR (⩾50% EBV miRNA positivity) and available tissue (*N*=6) and in six cases with little or no evidence of EBV miRNAs (⩽1 out of 12 amplifications with detectable EBV miRNA, or <10% EBV miRNA positivity) to establish whether EBV was localised to tumour cells. To detect and localise latent infection, *in situ* hybridisation (ISH) against *EBER* was performed on paraffin sections using the Bond system (Leica Microsystems, Buffalo Grove, IL, USA) (Lu *et al*, 2009). RNA preservation was examined in parallel by oligo-dT ISH as a quality control measure. To detect lytic infection, immunohistochemistry (IHC) was performed using antibody against lytic EBV protein BMRF1 (Research Diagnostics Inc., Flanders, NJ, USA). Infected Hodgkin's lymphoma was used as positive control for *EBER* ISH, and oral hairy leukoplakia was a positive control for BMRF1 immunostains. Localisation of signal to tumour *vs* reactive cells was interpreted by a pathologist. In addition, qPCR on extracted DNA was performed on an ABI 7500 using primers and a TaqMan probe (Applied Biosystems, Carlsbad, CA, USA) targeting EBV *BamH1W*, as previously published (Ryan *et al*, 2004). Efficacy of DNA extraction and amplification was examined by parallel amplification of the human *APOB* gene. DNA extracted from Namalwa cells was used as a standard and normaliser.

### Statistical analysis

#### Microarray

##### Class comparisons of lung adenocarcinoma *vs* squamous cell carcinoma

Differences between histologies in the expression of individual viral miRNAs were assessed using normalised data and two-sided *t*-tests. Given that we evaluated 32 viral miRNAs, we used an *α* level of 0.01 so that less than one miRNA would be expected to produce a significant result by chance. Multiple testing was accounted for in two ways: first, using the Benjamini and Hochberg method to estimate the false discovery rate (FDR) (Benjamini and Hochberg, 1995), and second using global permutation tests with 10 000 permutations, as previously described (Rotunno *et al*, 2010), to confirm overall significance of the expression profile differences. Analyses were also restricted to male smokers and adjusted by continuous age and stage (I, II, III, IV) to evaluate the robustness of results.

##### Correlation between microarray and qPCR

The qPCR ΔCt (cycle threshold) was calculated for each EBV miRNA by subtracting the average of the positive EBV miRNA Cts from the average Ct for miR-16. Cts >40 were considered missing (undetectable). Similar ratio measures were created for the microarray expression data by subtracting the miR-16 log-ratio from the microarray EBV miRNA log-ratios. The CORR procedure in SAS 9 (SAS Institute Inc., Cary, NC, USA) was used to calculate Spearman's rank-order correlation coefficient and test whether the correlation between the microarray ratio measures and qPCR ΔCt equalled zero for each EBV miRNA or pre-miRNA set. We also conducted sensitivity analyses to see if the definition of EBV positivity changed the results for correlation.

## Results

**Figure 1:**
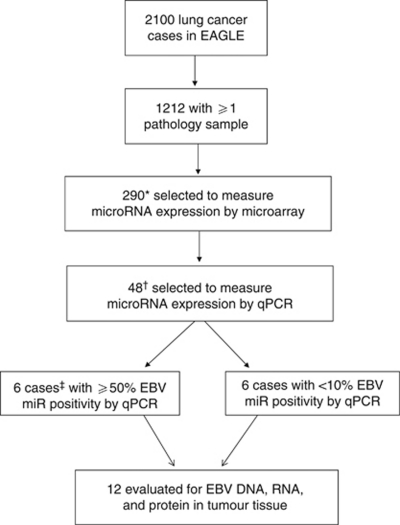
Description of case selection. ^*^Included all adenocarcinoma and squamous cell carcinoma cases who did not receive chemotherapy or radiation therapy before the study and had sufficient tissue available of non-mixed histologies. ^†^Included cases with sufficient RNA samples chosen for confirmation of viral and human miRNA expression as described by Landi *et al* (2010). ^‡^Of the 48 cases tested by qPCR, 7 cases were positive for EBV miRNAs (at least 6 out of 12 amplifications with detectable EBV miRNA). Of these seven cases, six had tissue available for follow-up analyses.

Of the 2100 lung cancer patients in EAGLE, 290 of the 656 cases with paraffin-embedded tissue blocks met the inclusion criteria, and 48 of those were tested for confirmation by qPCR. Among cases with available tissue, 6 with ⩾50% EBV miRNA positivity by qPCR and 6 with <10% EBV miRNA positivity by qPCR had follow-up testing for EBV (Figure 1). The median age for all 290 patients was 67 (range, 39–80). The distribution of lung adenocarcinoma and squamous cell carcinoma patients was similar to that seen in other populations (Ginsberg *et al*, 2007; Egleston *et al*, 2009; Fesinmeyer *et al*, 2010); adenocarcinoma cases were somewhat younger, as expected, and more likely to be female or never smokers than squamous cell carcinoma cases (Table 1). The majority of samples were from relatively early-stage (I–IIIA) tumours because surgical tissue samples were collected from resectable lesions.

Of the 42 viral miRNAs included on the miRNA microarray chip, 32 had <50% missing data (and 19 had <10% missing data) across all 290 samples. Most viral miRNAs appeared to be upregulated in adenocarcinoma compared with squamous cell carcinoma. The EBV miRNAs were the most common. Of 16 EBV miRNAs with <50% missing data, 9 (56%) strongly differentiated between adenocarcinoma and squamous cell carcinoma (parametric *P*-value <0.01; highlighted in bold in Table 2). Results were similar after adjusting for age and stage and restricting to male smokers, who made up the majority of cases (Supplementary Table 1).

Given this evidence that EBV miRNAs could distinguish between lung cancer histologies, we sought to confirm the microarray results by conducting qPCR for EBV pre-miRNAs BART1, BART2, and BHRF1–3 in a subset of cases (*n*=48) (Supplementary Table 2). All of the cases considered EBV miRNA positive (⩾50% qPCRs positive across all three EBV miRNAs) had an average Ct of <35 for BART1 (Supplementary Table 2). These EBV miRNA-positive cases included three patients with adenocarcinoma and four with squamous cell carcinoma. EBV miRNA positivity by qPCR did not distinguish squamous cell carcinoma from adenocarcinoma among the 48 cases tested by qPCR (χ^2^ *P*-value=0.6). Moreover, we found little correlation between microarray and qPCR for BART1, BART2, and BHRF1–3 expression (Spearman's correlation coefficients=0.12, 0.03, and 0.37 and *P*=0.53, 0.94, and 0.47, respectively). All correlation coefficients were low, suggesting substantial variation between the two platforms. Correlation coefficients remained low when we conducted sensitivity analyses using alternative definitions of EBV miRNA positivity. For example, when we compared the microarray log-ratio with any EBV miRNA detection by qPCR, the correlation coefficients were −0.02 (*P*=0.92) for BART1, −0.03 (*P*=0.90) for BART2, −0.21 (*P*=0.15) for BHRF1–3, and −0.01 (*P*=0.97) for miR-16.

To determine whether the EBV genome was present in tumour tissue from EBV miRNA-positive cases and whether the infection was located in tumour cells, rather than infiltrating lymphocytes, follow-up analyses were conducted in six cases with and six cases without strong evidence of EBV miRNAs (⩾50% positivity across all three EBV miRNAs) as measured by qPCR (Table 3). Although all EAGLE lung cancer diagnoses included in this study were confirmed by pathology reports from surgery, biopsy, or cytology samples (Landi *et al*, 2008), lung tumours are heterogeneous. Thus, 3 of the 12 cases evaluated for evidence of EBV in tumour tissue (one with sufficient evidence of EBV miRNAs and two without) did not have identifiable cancer in the remaining tissue samples used to test for *EBER* expression (Table 3). Of the remaining nine cases, none had detectable *EBER* expression in the malignant cells. One squamous cell carcinoma case had rare *EBER-*expressing lymphoid cells of the stroma but was not positive for any of the three EBV miRNAs by qPCR. In addition, none of the cases had lytic EBV infection as measured by IHC targeting the BMRF1 protein.

All 12 cases had amplifiable human *APOB* (median, 12 770; range, 2824–50 923), indicating successful DNA extraction. Two cases had EBV DNA detected at low copy number (Table 3). One was the case with stromal *EBER* expression, consistent with latent EBV infection in rare lymphocytes. The other case with evidence of EBV DNA also had detectable miRNA expression for BART1 in 4 out of 4 replicate qPCRs, BART2 in 3 out of 4 replicates, and BHRF1 in 1 out of 4 replicates. However, lack of *EBER* expression by ISH suggested that latent EBV was not localised to malignant cells or to surrounding lymphocytes. Nor did this tissue have detectable BMRF1, a protein indicative of replicative, lytic EBV infection. Taken together, these results suggest that the EBV DNA detected in this case did not reflect a typical malignancy-related EBV infection.

## Discussion

There is strong evidence that EBV miRNAs can contribute to carcinogenesis; EBV miRNAs have been detected in EBV-associated lymphomas and may affect immune surveillance by modulating cytotoxic lymphocyte cytokine networks (Xia *et al*, 2008). The EBV miRNAs can both promote the escape of EBV-infected cells from host immune surveillance by dysregulating viral and human gene expression (Lung *et al*, 2009) and interact with genes involved in apoptosis pathways, as supported by experimental evidence and computational prediction studies of miRNA/target pairs (Choy *et al*, 2008; Lagana *et al*, 2010). This evidence led us to test the hypothesis that viral miRNAs are present in tumour tissue of lung cancer patients.

Our initial microarray results were promising; most viral miRNAs were upregulated in adenocarcinoma compared with squamous cell carcinoma, supporting the hypothesis that infections may contribute to the development of adenocarcinoma. In addition, 56% of the analysed EBV miRNAs differentiated between adenocarcinoma and squamous cell carcinoma, supporting the hypothesis that EBV may be one such contributing infection. We tested a subset of samples using qPCR assays previously demonstrated to detect BART1, BART2, and BHRF miRNA in EBV-positive primary effusion lymphoma and Burkitt lymphoma cell lines or tissues but not EBV-negative samples like Kaposi sarcoma biopsies, immortalised virus-negative endothelial cell lines, or normal tonsil tissue (O’Hara *et al*, 2008, 2009a; Xia *et al*, 2008). However, the qPCR miRNA expression results from these prevalidated qPCR miRNA assays did not confirm the microarray results. When we further examined tumour tissue for EBV DNA, latently expressed EBV *EBER* RNA, and lytically expressed EBV BMRF1 protein in cases with and without strong qPCR-based evidence of EBV miRNAs, we found no evidence of traditional cancer-related EBV infection in the tumour tissue. Only one squamous cell carcinoma case had both *EBER* and EBV DNA detected, and the *EBER* expression in this case was localised to rare lymphocytes, rather than malignant epithelial cells. Although based on small numbers, these findings do not support the hypothesis that the EBV genome is present in malignant cells of EBV miRNA-positive cases. Furthermore, the findings suggest that EBV miRNA expression, including pre-miRNA and mature miRNA expression, might not correlate with conventional tissue-based measures of EBV infection in lung cancer, although it does in nasopharyngeal carcinoma (NPC) (Cosmopoulos *et al*, 2009).

The published epidemiologic evidence of EBV-associated lung cancer has been mixed. The EBV has been localised within the malignant cells of the rare LELC cases, particularly in Asian cases (Ho *et al*, 2006), but the EAGLE study did not include any LELC cases. The literature is less consistent for other lung histologies. Two previous serologic studies found a higher seroprevalence of several EBV antibodies in lung cancer cases compared with healthy controls (Desgranges and de-The, 1979; Roy *et al*, 1994). Among tissue-based studies, some studies have found 5–10% positivity by *EBER* ISH or EBV nuclear antigen IHC (Kasai *et al*, 1994; Wong *et al*, 1995; Grinstein *et al*, 2002), whereas others have found no positivity by *EBER* ISH (Conway *et al*, 1996; Kijima *et al*, 2001; Hayakawa *et al*, 2003; Chu *et al*, 2004; Lim *et al*, 2009) and generally low or no positivity by other markers (Chu *et al*, 2004). Although *EBER* ISH is considered the gold standard for detecting EBV-associated cancers because of its high abundance in latently EBV-infected cells, it is not a perfect measure (Delecluse *et al*, 2007). Thus, the current study took a more comprehensive approach than most previous tissue-based studies by using multiple tests to detect EBV. The PCR of *BamH1W* DNA represents a very sensitive assay of the EBV genome by virtue of targeting a reiterated segment of the EBV genome. In addition, the histochemical assays permit localisation of the virus to particular cell types by targeting the latent viral infection using *EBER* ISH and lytic viral infection using BMRF1 immunohistochemistry. Our tissue-based results support those of previous studies suggesting that EBV is not linked to lung cancer.

One possible explanation for the detection of EBV miRNAs without corresponding localisation of EBV infection to tumour cells is that our miRNA detection assays crossreacted with another target. Potential crossreaction is a general limitation of mature miRNA assays, as the target size is only ∼22 nucleotides. Another explanation is that the assays are so sensitive that even rare infected cells generate a positive result. Most adults carry EBV in about one in a million lymphocytes as a consequence of the fact that the virus persists for life in the human host following primary infection, and >90% of adults have serologic evidence of EBV infection (Rickinson and Kieff, 2007). Another explanation may be delivery of EBV miRNAs from infected lymphocytes to uninfected lung cells; recent cell line and mouse studies suggest that miRNAs, including EBV miRNAs, can be secreted in vesicles and delivered to other cells (Pegtel *et al*, 2010; Zhang *et al*, 2010). On the other hand, EBV miRNAs have been detected in *EBER*-positive NPC biopsies but not EBV-negative NPC cell lines (Cosmopoulos *et al*, 2009), suggesting that EBV miRNA expression is specific for EBV-infected cells. Even so, the potential for extracellular delivery of EBV miRNAs without accompanying infection should be explored.

We used qPCR, which is the recommended verification procedure for microarray miRNA profiling (Croce, 2009), and we further studied selected cases using microscopy-based histochemical analyses. Although both microarray and qPCR detected EBV mature miRNA or pre-miRNA expression in some lung cancer cases, results from these two platforms did not agree with each other. Such inconsistency between platforms is not uncommon (Koshiol *et al*, 2010). It could be because of technical differences in sensitivity and specificity of the different assays, the amount and quality of RNA available, or biological differences of the target molecules. By and large, pre-miRNA levels correlate with mature miRNA levels for EBV in lymphoma (Jiang *et al*, 2005; Lee *et al*, 2008; O’Hara *et al*, 2008, 2009b). However, it is possible that pre-miRNA levels may not correlate with mature miRNA levels in the lung cancer setting, which might explain the observed differences between the array platform that targets mature miRNAs and the qPCR platform that targets pre-miRNAs. In addition, although most miRNAs are stable, some do decay more quickly than others *in vivo* and *in vitro* (Bail *et al*, 2010), which potentially could lead to differences in the microarray and qPCR assays. Future studies should include a larger number of EBV miRNAs in methodological evaluations to refine EBV miRNA expression/detection technology to the point where it may be reliably employed in epidemiologic studies.

To our knowledge, this is the first study of viral miRNAs in lung cancer patients. By integrating multiple EBV detection methods and evaluating latent and lytic viral markers, we have provided a comprehensive assessment of EBV in the lung cancer cases we studied. The findings to date do not support a role for EBV in this disease.

## Figures and Tables

**Table 1:** Descriptive characteristics of EAGLE patients whose samples were used in miRNA microarray chip analysis^a^

**Characteristic**	**Adenocarcinoma (*n*=165)**	**Squamous cell carcinoma (*n*=125)**
Median age (range)	65 (39–79)	70 (42–80)
		
*Gender*		
Male	89 (54%)	122 (98%)
Female	76 (46%)	3 (2%)
		
*Smoking,* n *(%)*		
Current	70 (42%)	64 (51%)
Former^b^	61 (37%)	60 (48%)
Never	34 (21%)	1 (1%)
Average packs per day, median (25th–75th percentile)^c^	1.0 (0.75–1.0)	1.0 (1.0–1.5)
		
*Stage,* n *(%)*		
IA	32 (19%)	25 (20%)
IB	33 (20%)	27 (22%)
IIA	10 (6%)	4 (3%)
IIB	33 (20%)	38 (30%)
IIIA	32 (19%)	17 (14%)
IIIB	14 (8%)	13 (10%)
IV	11 (7%)	1 (1%)

Abbreviations: EAGLE=Environment And Genetics in Lung cancer Etiology; miRNA=microRNA.

a Adapted from Landi *et al* (2010).

b Former smokers were subjects who quit smoking ⩾6 months before the study.

c Data available for current and former smokers only.

**Table 2:** Expression of viral miRNAs in EAGLE patients with lung adenocarcinoma compared with squamous cell carcinoma

**MiRNA**	**Unique ID**	***P*-value^a^**	**FDR^b^**	**Permutation *P*-value**	**GM in AD^c^**	**GM in SQ^d^**	**Fold-change AD/SQ^e^**
hcmv-miR-UL70-3p	MIMAT0003343	1.00E−07	1.60E−06	<1e−07	0.99	0.68	1.46
**ebv-mir-BART8** f	MIMAT0003418	1.00E−07	1.60E−06	<1e−07	1.04	1.23	0.85
kshv-miR-K12-3	MIMAT0002193	2.00E−07	2.10E−06	<1e−07	1.62	1.18	1.37
**ebv-mir-BART4**	MIMAT0003412	7.00E−07	5.60E−06	<1e−07	0.47	0.35	1.35
**ebv-miR-BART1**	MIMAT0000999	1.20E−06	7.70E−06	<1e−07	0.22	0.16	1.39
**ebv-miR-BART6-3p**	MIMAT0003415	4.43E−05	2.36E−04	<1e−07	1.14	0.91	1.25
**ebv-miR-BART20-5p**	MIMAT0003719	1.04E−04	4.74E−04	1.00E−04	1.03	1.20	0.86
**ebv-mir-BART3** f	MIMAT0003410	4.47E−04	1.79E−03	5.00E−04	0.07	0.05	1.50
**ebv-mir-BART16**	MIMAT0003714	5.91E−04	2.10E−03	3.00E−04	1.09	1.19	0.91
**ebv-mir-BART13**	MIMAT0003424	7.35E−04	2.35E−03	<1e−07	1.20	1.03	1.17
kshv-miR-K12-8	MIMAT0002186	1.07E−03	3.12E−03	9.00E−04	1.57	1.29	1.21
sv40-miR-S1-5p	MIMAT0003344	1.25E−03	3.34E−03	1.40E−03	1.20	1.06	1.13
kshv-miR-K12-10a	MIMAT0002179	1.58E−03	3.89E−03	2.30E−03	0.82	0.63	1.30
kshv-miR-K12-10b	MIMAT0002180	2.01E−03	4.44E−03	2.10E−03	0.85	0.69	1.22
**ebv-miR-BHRF1-3**	MIMAT0000998	2.08E−03	4.44E−03	2.50E−03	0.15	0.12	1.29
kshv-miR-K12-1	MIMAT0002182	4.10E−03	7.73E−03	3.40E−03	1.04	1.13	0.92
hcmv-miR-US5-1	MIMAT0001579	4.11E−03	7.73E−03	3.60E−03	0.70	0.63	1.12
**ebv-mir-BART14**	MIMAT0003426	0.02	0.04	0.02	0.89	0.73	1.21
**ebv-mir-BART3**	MIMAT0003411	0.05	0.08	0.05	0.36	0.31	1.19
**ebv-miR-BART2**	MIMAT0001000	0.09	0.15	0.09	0.03	0.03	1.23
kshv-miR-K12-6-5p	MIMAT0002188	0.12	0.18	0.12	1.74	1.57	1.10
kshv-miR-K12-5	MIMAT0002190	0.13	0.18	0.12	1.16	1.22	0.95
hcmv-miR-US25-2-5p	MIMAT0001582	0.15	0.21	0.15	1.09	1.05	1.05
**ebv-mir-BART12**	MIMAT0003423	0.16	0.21	0.16	1.06	1.16	0.92
**ebv-miR-BART17-5p**	MIMAT0003715	0.22	0.28	0.22	1.27	1.23	1.04
**ebv-mir-BART5**	MIMAT0003413	0.23	0.28	0.23	2.91	2.67	1.09
hcmv-miR-UL112-1	MIMAT0001577	0.24	0.28	0.24	1.36	1.31	1.04
hcmv-miR-UL36-1	MIMAT0001576	0.27	0.31	0.27	1.08	1.12	0.96
hcmv-miR-US4	MIMAT0003341	0.35	0.38	0.35	1.07	1.09	0.98
**ebv-mir-BART10**	MIMAT0003420	0.36	0.38	0.35	1.18	1.11	1.06
kshv-miR-K12-2	MIMAT0002183	0.57	0.59	0.57	0.90	0.94	0.96
kshv-mir-K12-12	MIMAT0003712	0.87	0.87	0.87	1.18	1.17	1.01

Abbreviations: EAGLE=Environment And Genetics in Lung cancer Etiology; miRNA=microRNA; FDR=false discovery rate; GM=geometric mean; AD=adenocarcinoma; SQ=squamous cell carcinoma.

MiRNAs are sorted by the parametric *P*-value from the univariate test.

a Parametric *P*-value.

b FDR calculated by the method of Benjamini and Hochberg (1995).

c Geometric mean of miRNA expression in AD samples compared with the Epstein–Barr virus (EBV) reference sample.

d Geometric mean of miRNA expression in SQ samples compared with the EBV reference sample.

e Ratio of geometric mean ratios of miRNA expression in adenocarcinoma/squamous cell carcinoma.

f Denotes the antisense miRNA. Note: bold values indicate EBV miRNAs.

**Table 3:** Follow-up analyses in EAGLE cases with and without strong evidence of EBV miRNAs as measured by real-time quantitative PCR

**Patient**	**EBV miRNAs?^a^**	**ISH RNA control**	***EBER* expression in tumour**	***EBER* expression in stroma**	**IHC positivity for BMRF1**	**Copies of EBV DNA/100 000 cells**
1	Yes	1	NC^b^	No	No	0
2	Yes	2	No	No	No	0
3	Yes	0	No	No	No	0
4	Yes	0	No	No	No	0
5	Yes	ND^c^	No	No	No	17
6	Yes	3	No	No	No	0
7	No	1	NC^b^	No	No	0
8	No	1	NC^b^	No	No	0
9	No	3	No	No	No	0
10	No	3	No	No	No	0
11	No	1	No	No	No	0
12	No	0	No	Yes^d^	No	17

Abbreviations: EAGLE=Environment And Genetics in Lung cancer Etiology; EBV=Epstein–Barr virus; miRNA=microRNA; IHC=immunohistochemistry; ISH=*in situ* hybridisation.

a Yes=cases with ⩾50% EBV miRNA positivity (at least 6 out of 12 amplifications with detectable EBV miRNA); no=cases with <10% EBV miRNA positivity (⩽1 out of 12 amplifications with detectable EBV miRNA).

b NC=no cancer identified in tested specimen.

c ND=tissue lost from slide during ISH, and hence no RNA preservation control available.

d Positivity in lymphoid cells.
