# Supplementary material for: Epstein–Barr virus microRNAs and lung cancer
Source: Br J Cancer. 2011 Jun 7;105(2):320–6. doi: 10.1038/bjc.2011.221 (PMC3142804; doi:10.1038/bjc.2011.221)
Supplement: Supplementary Tables [file bjc2011221x1.doc]

**Supplemental Table 1. Comparison of viral microRNAs (miRNAs) between lung adenocarcinoma and squamous cell carcinoma with adjustment by age and stage in 290 EAGLE patients and restricting to 205 male smokers.**

|  |  |  | **Parametric p-value** | |
| --- | --- | --- | --- | --- |
| **MiRNA^†^** | **Unique ID** |  | **All cases (*n* = 290)** | **Male smokers (*n* = 205)** |
| hcmv-miR-UL70-3p | [MIMAT0003343](http://nciarray.nci.nih.gov/cgi-bin/clone_report.cgi?CRITERIA=clone&PARAMETER=MIMAT0003343:1a1) |  | 8.85E-08 | 1.64E-05 |
| **ebv-mir-BART8*** | [MIMAT0003418](http://nciarray.nci.nih.gov/cgi-bin/clone_report.cgi?CRITERIA=clone&PARAMETER=MIMAT0003418:2n14) |  | 1.42E-07 | 6.78E-05 |
| kshv-miR-K12-3 | [MIMAT0002193](http://nciarray.nci.nih.gov/cgi-bin/clone_report.cgi?CRITERIA=clone&PARAMETER=MIMAT0002193:2m4) |  | 1.91E-07 | 1.49E-05 |
| **ebv-mir-BART4** | [MIMAT0003412](http://nciarray.nci.nih.gov/cgi-bin/clone_report.cgi?CRITERIA=clone&PARAMETER=MIMAT0003412:2l8) |  | 8.38E-07 | 0.0003 |
| **ebv-miR-BART1** | [MIMAT0000999](http://nciarray.nci.nih.gov/cgi-bin/clone_report.cgi?CRITERIA=clone&PARAMETER=MIMAT0000999:2e11) |  | 1.03E-06 | 0.0002 |
| **ebv-miR-BART6-3p** | [MIMAT0003415](http://nciarray.nci.nih.gov/cgi-bin/clone_report.cgi?CRITERIA=clone&PARAMETER=MIMAT0003415:2f4) |  | 4.11E-05 | 0.002 |
| **ebv-miR-BART20-5p** | [MIMAT0003719](http://nciarray.nci.nih.gov/cgi-bin/clone_report.cgi?CRITERIA=clone&PARAMETER=MIMAT0003719:2l14) |  | 0.0001 | 0.002 |
| **ebv-mir-BART3*** | [MIMAT0003410](http://nciarray.nci.nih.gov/cgi-bin/clone_report.cgi?CRITERIA=clone&PARAMETER=MIMAT0003410:2h3) |  | 0.0005 | 0.002 |
| **ebv-mir-BART16** | [MIMAT0003714](http://nciarray.nci.nih.gov/cgi-bin/clone_report.cgi?CRITERIA=clone&PARAMETER=MIMAT0003714:2l15) |  | 0.0006 | 0.006 |
| **ebv-mir-BART13** | [MIMAT0003424](http://nciarray.nci.nih.gov/cgi-bin/clone_report.cgi?CRITERIA=clone&PARAMETER=MIMAT0003424:2g10) |  | 0.0008 | 0.003 |
| kshv-miR-K12-8 | [MIMAT0002186](http://nciarray.nci.nih.gov/cgi-bin/clone_report.cgi?CRITERIA=clone&PARAMETER=MIMAT0002186:2o2) |  | 0.001 | 0.003 |
| sv40-miR-S1-5p | [MIMAT0003344](http://nciarray.nci.nih.gov/cgi-bin/clone_report.cgi?CRITERIA=clone&PARAMETER=MIMAT0003344:1n20) |  | 0.001 | 0.003 |
| kshv-miR-K12-10a | [MIMAT0002179](http://nciarray.nci.nih.gov/cgi-bin/clone_report.cgi?CRITERIA=clone&PARAMETER=MIMAT0002179:2a2) |  | 0.002 | 0.001 |
| kshv-miR-K12-10b | [MIMAT0002180](http://nciarray.nci.nih.gov/cgi-bin/clone_report.cgi?CRITERIA=clone&PARAMETER=MIMAT0002180:2c2) |  | 0.002 | 0.001 |
| **ebv-miR-BHRF1-3** | [MIMAT0000998](http://nciarray.nci.nih.gov/cgi-bin/clone_report.cgi?CRITERIA=clone&PARAMETER=MIMAT0000998:2c11) |  | 0.002 | 0.02 |
| kshv-miR-K12-1 | [MIMAT0002182](http://nciarray.nci.nih.gov/cgi-bin/clone_report.cgi?CRITERIA=clone&PARAMETER=MIMAT0002182:2g2) |  | 0.004 | 0.01 |
| hcmv-miR-US5-1 | [MIMAT0001579](http://nciarray.nci.nih.gov/cgi-bin/clone_report.cgi?CRITERIA=clone&PARAMETER=MIMAT0001579:2a17) |  | 0.004 | 0.04 |
| **ebv-mir-BART14** | [MIMAT0003426](http://nciarray.nci.nih.gov/cgi-bin/clone_report.cgi?CRITERIA=clone&PARAMETER=MIMAT0003426:2j18) |  | 0.02 | 0.02 |
| **ebv-mir-BART3** | [MIMAT0003411](http://nciarray.nci.nih.gov/cgi-bin/clone_report.cgi?CRITERIA=clone&PARAMETER=MIMAT0003411:2d4) |  | 0.05 | 0.22 |
| **ebv-miR-BART2** | [MIMAT0001000](http://nciarray.nci.nih.gov/cgi-bin/clone_report.cgi?CRITERIA=clone&PARAMETER=MIMAT0001000:2g11) |  | 0.09 | 0.50 |
| kshv-miR-K12-6-5p | [MIMAT0002188](http://nciarray.nci.nih.gov/cgi-bin/clone_report.cgi?CRITERIA=clone&PARAMETER=MIMAT0002188:2c4) |  | 0.12 | 0.10 |
| kshv-miR-K12-5 | [MIMAT0002190](http://nciarray.nci.nih.gov/cgi-bin/clone_report.cgi?CRITERIA=clone&PARAMETER=MIMAT0002190:2g4) |  | 0.12 | 0.09 |
| hcmv-miR-US25-2-5p | [MIMAT0001582](http://nciarray.nci.nih.gov/cgi-bin/clone_report.cgi?CRITERIA=clone&PARAMETER=MIMAT0001582:2g17) |  | 0.15 | 0.48 |
| **ebv-mir-BART12** | [MIMAT0003423](http://nciarray.nci.nih.gov/cgi-bin/clone_report.cgi?CRITERIA=clone&PARAMETER=MIMAT0003423:2j22) |  | 0.16 | 0.68 |
| **ebv-miR-BART17-5p** | [MIMAT0003715](http://nciarray.nci.nih.gov/cgi-bin/clone_report.cgi?CRITERIA=clone&PARAMETER=MIMAT0003715:2j3) |  | 0.22 | 0.48 |
| **ebv-mir-BART5** | [MIMAT0003413](http://nciarray.nci.nih.gov/cgi-bin/clone_report.cgi?CRITERIA=clone&PARAMETER=MIMAT0003413:2n21) |  | 0.22 | 0.25 |
| hcmv-miR-UL112-1 | [MIMAT0001577](http://nciarray.nci.nih.gov/cgi-bin/clone_report.cgi?CRITERIA=clone&PARAMETER=MIMAT0001577:2m15) |  | 0.24 | 0.25 |
| hcmv-miR-UL36-1 | [MIMAT0001576](http://nciarray.nci.nih.gov/cgi-bin/clone_report.cgi?CRITERIA=clone&PARAMETER=MIMAT0001576:2k15) |  | 0.27 | 0.67 |
| hcmv-miR-US4 | [MIMAT0003341](http://nciarray.nci.nih.gov/cgi-bin/clone_report.cgi?CRITERIA=clone&PARAMETER=MIMAT0003341:1a5) |  | 0.35 | 0.75 |
| **ebv-mir-BART10** | [MIMAT0003420](http://nciarray.nci.nih.gov/cgi-bin/clone_report.cgi?CRITERIA=clone&PARAMETER=MIMAT0003420:2j5) |  | 0.36 | 0.56 |
| kshv-miR-K12-2 | [MIMAT0002183](http://nciarray.nci.nih.gov/cgi-bin/clone_report.cgi?CRITERIA=clone&PARAMETER=MIMAT0002183:2i2) |  | 0.57 | 0.38 |
| kshv-mir-K12-12 | [MIMAT0003712](http://nciarray.nci.nih.gov/cgi-bin/clone_report.cgi?CRITERIA=clone&PARAMETER=MIMAT0003712:2l5) |  | 0.87 | 0.63 |

*Denotes the antisense miRNA.

^†^MiRNAs are sorted by the parametric p-value from the age- and stage-adjusted analysis among all patients.

**Supplemental Table 2. Microarray chip and quantitative real-time PCR (qPCR) results for selected EAGLE lung cancer cases.**

*Percent of runs with detectable miRNA expression out of 12 total runs, 4 for each of the 3 EBV miRNAs.

†This patient had no tissue available for follow-up.
